# Risk of hypertension in cancer patients treated with abiraterone: a meta-analysis

**DOI:** 10.1186/s40885-019-0116-x

**Published:** 2019-06-01

**Authors:** Xiaolei Zhu, Shenhong Wu

**Affiliations:** 1 Division of Primary Care, State University of New York at Stony Brook, 205 North Belle Mead Road, East Setauket, Stony Brook, NY 11733 USA; 2 Division of Hematology and Oncology, Department of Medicine, School of Medicine, State University of New York at Stony Brook, 3 Edmund D. Pellegrino Rd, Stony Brook, NY 11794-9447 USA; 3 Division of Hematology and Oncology, Department of Medicine, Northport VA Medical Center, Northport, NY USA

**Keywords:** Abiraterone, Prostate cancer, Hypertension, Adverse event, Risk

## Abstract

**Background:**

Hypertension is one of the major side effects associated with abiraterone in the treatment of advanced prostate cancer. The specific contribution of abiraterone to hypertension has not been defined. We performed a systematic review and meta-analysis of randomized clinical trials to determine its overall risk.

**Methods:**

Databases including Pubmed (up to July 2018) and Google scholar (up to July 2018) were searched to identify relevant studies. Eligible studies were prospective randomized clinical trials with prostate cancer treated with abiraterone and prednisone. The incidence and relative risk (RR) of hypertension was calculated using random-effects or fixed-effects model depending on the heterogeneity of included studies.

**Results:**

A total of five studies including 5445 patients were selected for analysis. Among patients receiving abiraterone, the overall incidences of all grade and high grade (grade 3 and 4) were 21.9% (95% CI: 13.6–33.2%) and 10.2% % (95% CI: 6.9–11.6%). Abiraterone was associated with a significantly increased risk of hypertension of all grade with a relative risk of 1.80 (95% CI: 1.47–2.19%, *p* < 0.001) and high grade with a relative risk of 2.11 (95%CI: 1.66–2.68%, p < 0.001) in comparison with controls. The risk of hypertension may be affected by concurrent use of prednisone with 5 mg daily is associated with higher incidence than that of prednisone 5 mg twice daily (32.4% vs 16.5%).

**Conclusion:**

There is a significant increase of developing hypertension in prostate cancer patients treated with abiraterone. Appropriate monitoring and management is strongly recommended to reduce the risk of cardiovascular events and treatment interruptions.

## Introduction

Abiraterone acetate, the prodrug of abiraterone, is a selective inhibitor of androgen biosynthesis that potently blocks cytochrome P450 c17 (CYP17), a critical enzyme in testosterone synthesis, thereby blocking androgen synthesis by the adrenal glands and testes, as well as within the prostate tumor [1–4]. The use of abiraterone in combination with prednisone has been shown to be effective in treating advanced prostate cancer and prolonging overall survival of these patients [5–8]. Recently, abiraterone received FDA approval for the treatment of castration resistant or sensitive metastatic prostate cancer due to its clinical benefit.

As with many other therapeutic agents, the use of abiraterone is associated with significant side effects. Fatigue and hot flash were the most common adverse events associated with abiraterone. In addition, mineralcorticoid excess such as hypokalemia, hypertension and fluid overload is a major side effect observed in clinical trials [6, 9]. The concurrent use of prednisone is recommended to mitigate adverse events related to the mineralocorticoid excess. However, hypertension is consistently a major side effect observed in the clinical trials ranging even with the use of prednisone [10]. The recognition and management of hypertension is an important issue in this patient group since uncontrolled hypertension can lead to serious cardiovascular risk and treatment interruptions. In fact, earlier study showed increased risk of cardiac events in patient treated with abiraterone [11]. More importantly, the use of abiraterone can be associated with fatal cardiac events including heart failure [10]. In addition, the issue may be more prominent in elderly patients who likely has more cardiovascular risk factors and the age-related risk of vascular disease [12].

Because of the limited number of patients in each trial, the overall incidence and risk of hypertension with abiraterone is unclear. The incidences of hypertension varied widely ranging from 3.3 to 36.7% in clinical trials [6, 7, 9, 13, 14]. In addition, the exact contribution of abiraterone to hypertension is not clearly defined due to confounding factors such as comorbidities and concurrent medications. We have conducted a systematic review of the literature and a meta-analysis of randomized controlled trials in which the combination of abiraterone with prednisone was compared to controls to evaluate this risk. In addition, due to the importance of glucocorticoids in the treatment and slightly different dose used in clinical trials, subgroup analysis of hypertension risk with different prednisone dose was also analyzed.

## Methods

### Data source

An independent review of citations from Pubmed database up to July 2018 was conducted. The search included key words “abiraterone”, “prostate cancer”, and was limited to randomized clinical trials. In addition, Google Scholar citation was searched and manually reviewed to ensure that there were no additional randomized clinical trials. The computer search was supplemented with manual review of the retrieved articles to make sure they meet the inclusion criteria and disregard the duplications. With multiple publications within the same clinical trial, the publication from the most recent, complete and updated version was included in this meta-analysis. Data extracted from the selected trials included details on study design, patient characteristics, treatment information, results, and follow-up from these selected trials. The search, selection, and exclusion of the studies were performed by one of the authors (X.Z.). We also reviewed data and information from the updated package insert of abiraterone.

### Study selection

Abiraterone in combination with prednisone has been approved for the use in patients with advanced prostate cancer, thus it has practical implications to evaluate the risk of hypertension with the combination of abiraterone with prednisone. In order to exclude the influence of comorbidities such as hypertension commonly seen in the prostate cancer patients with elderly population, we included only randomized controlled phase II and phase III trials to determine the particular contribution of the treatment to the hypertension. Trials that met the following criteria were chosen for analysis: 1) prospective clinical trials in prostate cancer patients; 2) random assignment of participants to the treatment with abiraterone with prednisone or controls: 3) data available for the events or incidences of hypertension. The quality of the selected trials was assessed by the 7-iem Jadad scale including randomization, double-blinding, and withdrawals as described previously [15].

### Clinical end points

The clinical end points were extracted from the safety profile in each trial. Hypertension was recorded according to versions III of the Common Terminology Criteria for Adverse Events (CTCAE) of National Cancer Institute (http://ctep.cancer.gov/reporting/ctc_archive.html) [16]. Grade I, asymptomatic, transient (< 24 h) increase of blood pressure by > 20 mmHg (diastolic) or to > 150/100 mmHg if previously within normal limit (WNL), intervention not indicated; grade II, recurrent or persistent (> 24 h) or symptomatic increase by > 20 mmHg (diastolic) or to > 150/100 mmHg if previously WNL, monotherapy may be indicated; grade III, requiring more than one drug or more intensive therapy than previously; grade IV, life-threatening consequences (e.g., hypertensive crisis). We have included the incidence of hypertension of grade I and above for our analysis.

### Statistical analysis

All statistical analysis was performed using version 2 of the Comprehensive MetaAnalysis program (Biostat, Englewood, NJ). We have previously described the use of the program for the risk analysis of serious adverse events with targeted therapy in cancer patients [17–19]. The numbers of patients with hypertension, both all-grades and high-grades (grade 3 & 4), were summarized from the data extracted from all patients receiving the combination of abiraterone (starting dose 1000 mg daily) and prednisone (starting dose 5 or 10 mg daily) in those clinical trials included for analysis. For each trial, the proportion of patients with hypertension was calculated, and its 95% confidence interval was derived. The relative risk (RR) of hypertension among patients assigned to abiraterone/prednisone was also calculated, compared only with those assigned to control treatment in the same trial.

For meta-analysis, both the fixed-effects model (weighted with inverse variance) and the random-effects model were considered [20]. For each meta-analysis, the Cochran’s Q statistic was first calculated to assess the heterogeneity of the included trials. For *p*-value less than 0.1, the assumption of homogeneity was deemed invalid [21], and the random-effects model was used only after significant efforts were taken to explore the possible reasons for the heterogeneity. Otherwise, data were evaluated using both the fixed-effects model and the random-effects models. A two-tailed *p*-value of less than 0.05 was judged as statistically significant. All the statistical analyses were performed by one of the authors (S.W.).

## Results

### Search results

**Fig. 1:**
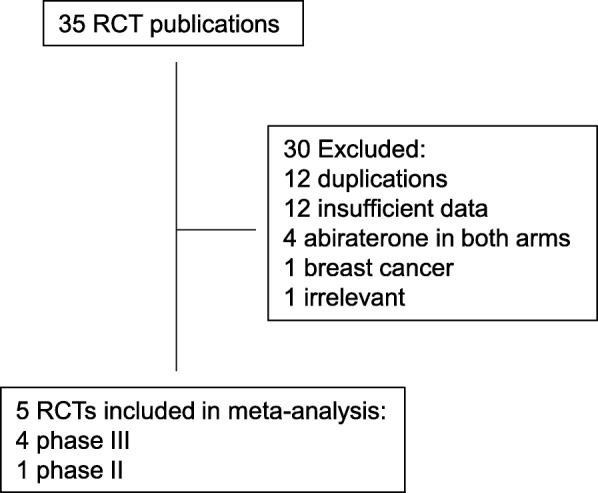
Selection of randomized controlled trials (RCT) included in the meta-analysis

Our search yielded a total of 35 articles on the clinical trials of abiraterone in prostate cancer from the literature. After excluding review articles, phase I studies, single-arm phase II studies, case reports, meta-analyses, and observational studies (Fig. 1), we selected 5 randomized controlled trials, [6, 7, 9, 13, 14] including 4 phase III studies and one phase II study, which fulfilled our inclusion criteria; and their characteristics are listed in Table 1. Of note, for a trial with multiple publications of results derived from the same patient population, the paper that has the most updated data was selected. All the trials selected were sponsored by Jassen Research & Development (formerly Ortho Biotech Oncology Research and Development Unit of Cougar Biotechnology) and other organizations.

**Table 1:** Characteristics of clinical trials and patients included in the meta-analysis

Trial name	Design	Total enrollment number	Intervention	Control	Study quality
James et al., 2017 [7]	Open label-phase III	1917	Abiraterone prednisone 5 mg qd ADT	ADT alone	4
Fizazi et al., 2017 [13]	Double-blind phase III	1199	Abiraterone Prednisone 5 mg qd ADT	ADT + placebo	5
Ryan et al., 2015 [9]	Double-blind phase III	1088	Abiraterone Prednisone 5 mg bid	Placebo Prednisone 5 mg bid	5
Taplin et al., 2014 [14]	Open label phase II	56	Abiraterone Prednisone 5 mg qd ADT	ADT alone	3
Fizazi et al., 2012 [6]	Double-blind phase III	1185	Abiraterone Prednisone 5 mg bid	Placebo Prednisone 5 mg bid	5

*Abbreviations*: *ADT* Androgen deprivation therapy. qd, once a day; bid, twice a day

### Study quality

Randomized treatment allocation sequences were generated in all trials. Three trials were double-blinded and placebo-controlled [6, 9, 13], and the rest of the trials had active treatment control. Hypertension as the primary endpoint of the study was assessed and recorded according to NCI-CTC version 3 in these trials (Table 1). Follow-up time was adequate for each trial. The Jadad score was listed for each trial in Table 1, and the average score was 3.5 with a range between 3 and 5. Therefore, the overall quality of all the trials was good.

### Patients

A total of 5445 prostate cancer patients were available for analysis, with 2909 patients were treated with the combination of abiraterone with prednisone. Baseline characteristics of these patients from the five randomized clinical trials were listed in Table 1. Baseline Hypertension was not specified in the pre-existing condition in these trials. All the patients in these trials are castration resistant or sensitive metastatic prostate cancer. In the phase II trial, only data up to 12 weeks was used since all patients used abiraterone after 12 weeks.

### Incidence of all-grade hypertension

**Fig. 2:**
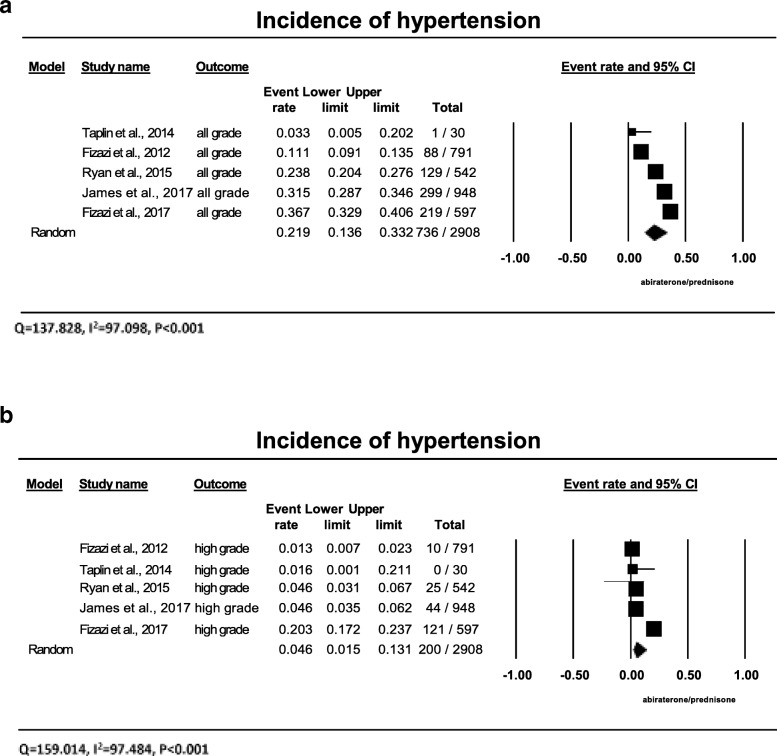
Annotated forest plot for meta-analysis of the incidence of hypertension in cancer patients who received abiraterone. The summary incidences of all-grade (**a**) and high-grade (**b**) hypertension are calculated using a random-effects model. The incidences and 95% confidence intervals for each study and the final combined result are displayed numerically on the left and graphically as a forest plot on the right. Under study name, the first author’s name and the publication year were used to represent each trial. The size of the squares is directly proportional to the amount of information available. For individual trials: filled-in square, incidence; lines, 95% confidence interval; diamond plot, overall results of the included trials

A total of 2908 patients with prostate cancer from 5 trials received the combination of abiraterone and prednisone with the data of all-grade hypertension available for analysis. The incidences of all-grade hypertension ranged from 3.3 to 36.7%, with the lowest incidence observed in the phase II clinical trial with smallest patient number of only 56 patients [14], and the highest incidence in patients with castration-sensitive disease receiving 5 mg daily prednisone [13]. Meta-analysis showed that heterogeneity (Q = 137.828, I2 = 97.098, *P* < 0.001) exists among these trials. As analyzed by the random-effects model, the summary incidence of all-grade hypertension was 21.9% (95% CI: 13.6–33.2%) among those patients receiving the combination of abiraterone and prednisone from all these trials (Fig. 2a). We didn’t find compelling reasons to exclude any of trials.

### Incidence of high-grade hypertension

High-grade hypertension requires more than one drug or more intense therapy than previously (grade 3), or is associated with life-threatening consequences (grade 4). It can result in significant morbidity, dose reduction or discontinuation of abiraterone. A total of 2908 patients from all 5 clinical trials received abiraterone and prednisone with the data of high-grade hypertension available for analysis. The incidence of grade 3 or 4 hypertension ranged between 1.3 and 20.3%, with the lowest observed in the phase III trial in patients with castration-refractory prostate cancer receiving prednisone 10 mg daily [6], and the highest incidence again seen in the phase III randomized trial in patients with castration-sensitive prostate cancer receiving 5 mg of prednisone [13]. The summary incidence of high-grade hypertension was 4.6% (95% CI: 1.5–13.1%) from these studies, as determined by the random-effects model (Fig. 2b).

### The influence of prednisone dose on the risk of hypertension in patients taking abiraterone

Due to the heterogeneity of the above analyses, we explored its underlying causes. Abiraterone is usually given with small dose prednisone to alleviate side effects. In these clinical trials, either 5 mg or 10 mg daily was used in these prostate cancer patients respectively. We have performed subgroup analysis of hypertension risk with abiraterone among patients taking abiraterone 5 mg prednisone daily versus 5 mg prednisone twice a day. The incidence of all-grade hypertension with abiraterone was 16.5% (95% CI: 7.5–32.7%) among patients taking prednisone 5 mg twice a day, comparing to 32.4% (25.4–40.2%) among patients taking prednisone 5 mg once daily. However, the difference didn’t reach a statistic significance (*P* = 0.07).

Similarly, there was a difference for high-grade hypertension between 10 mg and 5 mg daily prednisone. The incidence of high-grade hypertension with abiraterone was 2.5% (95% CI: 0.7–8.6%) among patients taking prednisone 5 mg twice a day, comparing to 7.5% (1.8–26.2%) among patients taking prednisone 5 mg once daily. However, the difference didn’t reach a statistic significance (*P* = 0.25).

### Relative risk of hypertension

**Fig. 3:**
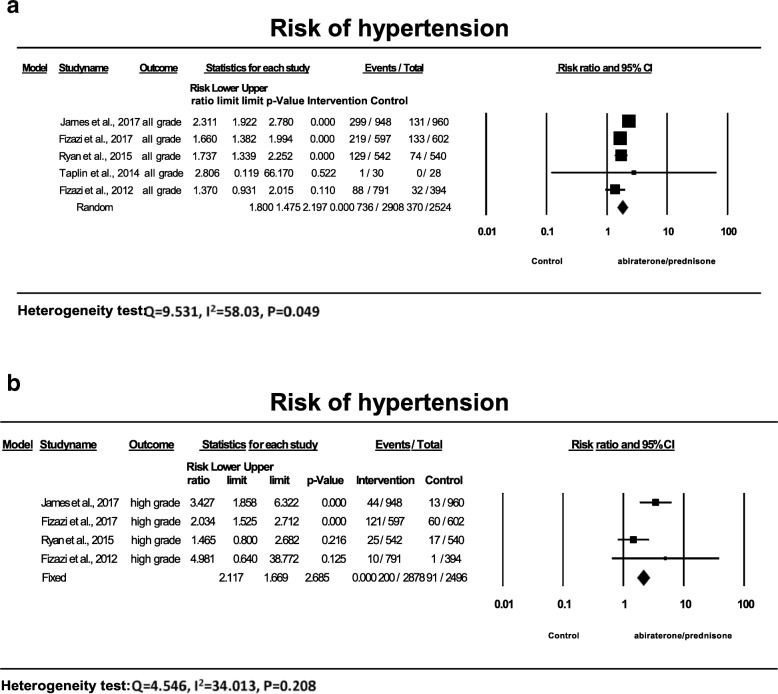
Relative risk of hypertension associated with abiraterone versus control. The summary relative risk (RR) was calculated using the random-effects model. RR and 95% confidence intervals for each study and the final combined result are displayed numerically on the left and graphically as a forest plot on the right. Under study name, the first author’s name was used to represent each trial. The size of the squares is directly proportional to the amount of information available. For individual trials: filled-in square, RR; lines, 95% confidence interval; diamond plot, overall results of the included trials

In order to assess the specific contribution of abiraterone to the development of hypertension and exclude the influence of other factors in these patients with comorbidities and concurrent medications, we have determined a relative risk of hypertension (RR) associated with abiraterone in comparison with controls (3 trials used ADT, and 2 trials prednisone). As shown in Fig. 3, the summary RR was 1.80 (95% CI: 1.48–2.20, *P* < 0.001) for all-grade hypertension (Fig. 3a), and 2.12 (95% CI: 1.70–2.69, P < 0.001) for high-grade hypertension (Fig. 3b). Thus, abiraterone with prednisone was associated with a significant increased risk of hypertension in patients with prostate cancer.

Subgroup analyses showed abiraterone/prednisone with ADT significantly increased the risk of all-grade hypertension with an RR of 1.97 (95%CI: 1.46–2.64) in comparison with ADT alone, and an RR of 1.61 (95%CI: 1.30–2.0) in comparison with prednisone/placebo/ADT. No significant difference was found between ADT and prednisone as a control for all-grade hypertension (*P* = 0.29).

## Discussion

This study has determined the overall risk of hypertension associated with the use of abiraterone and prednisone in patients with advanced prostate cancer. We have demonstrated a high incidence of all-grade hypertension (21.9, 95% CI: 13.6–33.2%) and high-grade hypertension (10.2, 95% CI: 6.9–11.5%) associated with abiraterone and prednisone. In addition, the combination significantly increased the risk of hypertension in comparison with controls with RR 1.80 (95% CI: 1.47–2.19, *P* < 0.001) for all-grade and 2.11 (95% CI: 1.66–2.68) for high-grade. Adequate monitoring and aggressive management of moderate hypertension is essential for many of these patients, as hypertension is an independent risk factor for cardiovascular events. In fact, abiraterone-based treatment is associated with significantly increased risk of cardiac disorder [11]. Also, poorly controlled hypertension associated with abiraterone may lead to cardiac deaths [10]. In addition, severe hypertension has led to the permanent discontinuation of abiraterone. Because it has been shown to be effective in patients with advanced age (over 75 years of age) [22], the use of abiraterone with prednisone may be common in elderly patients. With a higher risk of cardiovascular events in elderly patients, early recognition and effective management becomes more important, not only at oncologist’s office, but also in primary care setting.

The association of abiraterone with hypertension may have multiple mechanisms including increased mineral corticoid production, reduced androgen synthesis, and anti-cancer effect. It is likely directly related with cytochrome P450 17A1 (CYP17A1) inhibition resulting in significant suppression of androgen and cortisol synthesis. The suppression of cortisol leads to increase of ACTH which in turn causes mineral corticoid excess. Addition of steroid reverses the side effects mediated by the elevated mineral corticoids. Even though addition of dexamethasone 0.5 mg /day which is equivalent to 3 mg of prednisone/day is sufficient to suppress plasma ACTH to below the lower limit of sensitivity in patients studied before [3], Abiraterone with prednisone 5 mg daily or twice daily is still associated with significant hypertension risk. This is likely due to incomplete suppression of ACTH. Our study has shown a trend favoring the idea that abiraterone with prednisone 5 mg bid is associated with less risk of hypertension comparing to patients on abiraterone with 5 mg prednisone daily, and supported the notion that even 5 mg daily of prednisone may not sufficiently suppress ACTH elevation with abiraterone.

The association of abiraterone with hypertension may be also related to its anti-androgen effect due to reduced androgen synthesis. Hypertension associated with anti-androgen therapy is an emerging issue in patients receiving these therapeutic agents and a part of metabolic syndrome with diabetes and obesity [23–25]. In addition to abiraterone, several other androgen-blocking agents such as enzalutamide, and apalutamide have been associated with the development of hypertension (Table 2) [26, 27]. Our study demonstrated that the combination of abiraterone and prednisone is associated with a significant increased risk of all-grade hypertension with an incidence of 21.9% (95% CI: 13.6–33.2%) and RR of 1.80 (95% CI: 1.47–2.19, *P* value < 0.0001). Taken together, it appears that the risks of hypertension associated with these androgen inhibitors are considerable.

**Table 2:** Risks of hypertension with abiraterone

	MOA	Incidence (95% CI)	Relative risk (95% CI)	References
abiraterone and prednisone	Androgen biosynthesis (CYP-17 inhibitor)	21.9% (13.6–33.2%)	1.80 (1.47–2.19)	This study
enzalutamide [26]	Anti-Androgen receptor	14%	3.41	Package insert ^a^
leuprolide [34]	Androgen biosynthesis (LHRH analogs)	6.6%	NA	Package insert
firmagon [35]	Androgen biosynthesis (LHRH antagonist)	7%	NA	Package insert

*Abbreviations*: *MOA* Mechanism of action, *LHRH* Luteinizing hormone releasing hormone. ^a^ The incidence and RR were calculated from PREVAIL trial

The association of abiraterone may also partially caused by its anti-tumor effect. It is well-known that progression of advanced cancer can decrease blood pressure due to increased VEGF secretion, weight loss, and anorexia. Anti-VEGF therapy can increase blood pressure in cancer patients [28–30]. Response of prostate cancer to abiraterone would decrease VEGF production from tumors and improve food intake/weight gain, and thus elevate blood pressure.

Management of abiraterone-associated hypertension is not well described. According to the manufacturer package insert regarding warnings and precautions, hypertension as well as hypokalemia and fluid retention may be caused by abiraterone as a consequence of increased mineralocorticoid levels resulting from CYP17 inhibition. Monitor patients for hypertension, hypokalemia, and fluid retention at least once a month. Control hypertension before and during treatment with abiraterone. Closely monitor patients whose underlying conditions might be compromised by increases in blood pressure, hypokalemia or fluid retention such as those with heart failure, recent myocardial infarction, cardiovascular disease or ventricular arrhythmia.

There is no standard management of abiraterone treatment related hypertension. Gill and Gaston et al. showed comparable side effects among patients treated with abiraterone and epleronone vs abiraterone and prednisone [31]. In addition, due to additional side effects of hypokalemia and fluid retention, epleronone is a choice due to its diuretic effect and the ability to reduce potassium wasting [31]. However, in the similar category of epleronone, spironolactone is not recommended due to its potential side effect of promoting prostate tumor growth by compromising the therapeutic effect of abiraterone [32]. Though prednisone 5 mg twice daily with abiraterone seems to have less hypertension effect comparing to prednisone 5 mg daily with abiraterone, however long term use of steroids may be still associated with significant side effect even with such small dose for some patients. In cases of severe or persistent hypertension, despite institution of antihypertensive therapy, temporary or permanent discontinuation of abiraterone should be considered. Abiraterone is a strong inhibitor of CYP2D6 and moderate inhibitor of CYP3A4. Drug-drug interaction needs to be monitored closely. Metoprolol is a very common medication in elderly patients with cardiovascular risk. Abiraterone is shown to increase metoprolol effect due to strong CYP2D6 inhibition. According to the manufacture insert, recommend alternative drug to avoid metoprolol toxicity. If metoprolol is needed in patients treated with abiraterone, dose reduction may be needed. Thus, increasing metoprolol dose or starting metoprolol is not recommended for treating abiraterone related hypertension. Strong CYP3A4 inducers such as ST. John’s Wort, phenytoin, carbamazepine and etc., should be avoided since they may decrease serum concentration of abiraterone.

The present study has some limitations. As with any meta-analysis, the results described here are affected by the limitation of individual clinical trial that was included for the analysis. Firstly, these trials may have underestimated the incidence of hypertension associated with abiraterone because of the imperfection of the National Cancer Institute Common Toxicity Criteria Version III to record adverse events [33]. In this version of toxicity criteria, patients were considered hypertensive only if diastolic pressure increased > 20 mmHg, or BP was > 150/100 mmHg. This grading criteria would have missed many patients with hypertension according to the standard criteria for the diagnosis of hypertension (130/80 mmHg). Also, the grading criteria does not differentiate between grade 2 and 3 hypertensions well, and our results of high-grade hypertension could be affected by the overlap between these two grades. Secondly, the prevalence of baseline hypertension was not described in these trials. This may lead to an overestimation of the incidence of hypertension with abiraterone. However, the presence of baseline hypertension may likely to be low, about 0–13.4% as estimated from the patients receiving placebo in control groups. We have minimized the likelihood of bias by calculating relative risk using randomized controlled clinical trials for direct comparison with and without abiraterone. In addition, there appears no significant variation in the incidence of baseline hypertension in patients with metastatic cancers as reflected in the control arms of many clinical trials. Finally, patients in this study were a selected group with metastatic cancers involved in clinical trials. The results were observed mainly in academic centers and major research institutes, and may not entirely apply to cancer patients treated in the community.

## Conclusion

This study has demonstrated that the combination of abiraterone with prednisone is associated with significantly increased risk of all-grade and high-grade hypertension in prostate cancer patients. The risk may vary with the dose of prednisone. It is important for physicians and patients to recognize the risk of all-grade hypertension, but also to appreciate the risk of serious hypertension. Early detection and effective management may allow safe use of abiraterone, reducing cardiovascular risk and treatment interruption/discontinuation and improving the overall outcome of these patients.
